# Supplementary material for: Public involvement and engagement in big data research: protocol for a scoping review and a systematic review of delivery and effectiveness of strategies for involvement and engagement
Source: BMJ Open. 2021 Aug 19;11(8):e050167. doi: 10.1136/bmjopen-2021-050167 (PMC8378392; doi:10.1136/bmjopen-2021-050167)
Supplement: Author's manuscript [file bmjopen-2021-050167.draft_revisions.pdf]

BMJ Open is committed to open peer review. As part of this commitment we make the peer review history of every article we publish publicly available.

When an article is published we post the peer reviewers' comments and the authors' responses online. We also post the versions of the paper that were used during peer review. These are the versions that the peer review comments apply to.

The versions of the paper that follow are the versions that were submitted during the peer review process. They are not the versions of record or the final published versions. They should not be cited or distributed as the published version of this manuscript.

BMJ Open is an open access journal and the full, final, typeset and author-corrected version of record of the manuscript is available on our site with no access controls, subscription charges or pay-per-view fees (http://bmjopen.bmj.com).

If you have any questions on BMJ Open's open peer review process please email info.bmjopen@bmj.com

BMJ Open

Public involvement and engagement in big data research: protocol for a scoping review and a systematic review of delivery and effectiveness of strategies for involvement and engagement.

Journal:	*BMJ Open*
Manuscript ID	bmjopen-2021-050167
Article Type:	Protocol
Date Submitted by the Author:	15-Feb-2021
Complete List of Authors:	Teodorowski, Piotr; University of Liverpool<br>Jones, Elisa; University of Liverpool<br>Tahir, Naheed; ARC NWC Public Adviser<br>Ahmed, Saiqa; ARC NWC Public Adviser<br>Frith, Lucy; University of Liverpool
Keywords:	PUBLIC HEALTH, QUALITATIVE RESEARCH, MEDICAL ETHICS

SCHOLARONE™
Manuscripts

BMJ

I, the Submitting Author has the right to grant and does grant on behalf of all authors of the Work (as defined in the below author licence), an exclusive licence and/or a non-exclusive licence for contributions from authors who are: i) UK Crown employees; ii) where BMJ has agreed a CC-BY licence shall apply, and/or iii) in accordance with the terms applicable for US Federal Government officers or employees acting as part of their official duties; on a worldwide, perpetual, irrevocable, royalty-free basis to BMJ Publishing Group Ltd ("BMJ") its licensees and where the relevant Journal is co-owned by BMJ to the co-owners of the Journal, to publish the Work in this journal and any other BMJ products and to exploit all rights, as set out in our licence.

The Submitting Author accepts and understands that any supply made under these terms is made by BMJ to the Submitting Author unless you are acting as an employee on behalf of your employer or a postgraduate student of an affiliated institution which is paying any applicable article publishing charge ("APC") for Open Access articles. Where the Submitting Author wishes to make the Work available on an Open Access basis (and intends to pay the relevant APC), the terms of reuse of such Open Access shall be governed by a Creative Commons licence – details of these licences and which Creative Commons licence will apply to this Work are set out in our licence referred to above.

Other than as permitted in any relevant BMJ Author's Self Archiving Policies, I confirm this Work has not been accepted for publication elsewhere, is not being considered for publication elsewhere and does not duplicate material already published. I confirm all authors consent to publication of this Work and authorise the granting of this licence.

For peer review only

Title: Public involvement and engagement in big data research: protocol for a scoping review and a systematic review of delivery and effectiveness of strategies for involvement and engagement.

Authors:

Piotr Teodorowski, Institute of Population Health, University of Liverpool, p.teodorowski@liverpool.ac.uk ORCID: 0000-0003-2172-8298

Elisa Jones, Institute of Population Health, University of Liverpool, elisa.jones@liverpool.ac.uk

Naheed Tahir, ARC NWC Public Adviser, naheedtahir33@hotmail.co.uk

Saiqa Ahmed, ARC NWC Public Adviser, saiqa75@hotmail.co.uk

Lucy Frith, Institute of Population Health, University of Liverpool, frith@liverpool.ac.uk, ORCID: 0000-0002-8506-0699

Corresponding author: Piotr Teodorowski

Word count: 2742

For peer review only

Public involvement and engagement in big data research: protocol for a scoping review and a systematic review of delivery and effectiveness of strategies for involvement and engagement.

Abstract

Introduction: Big data research has grown considerably over the last two decades. This presents new ethical challenges around consent, data storage and anonymisation. Big data research projects require public support to succeed and it has been argued that one way to achieve this is through public involvement and engagement. To better understand the role public involvement and engagement can play in big data research, we will review the current literature. This protocol describes the planned review methods.

Methods and analysis: Our review will be conducted in two stages. In the first stage, we will conduct a scoping review using Arksey and O'Malley (2005) methodology to comprehensively map current evidence on public involvement and engagement in big data research. Databases (CINAHL, Health Research Premium Collection, PubMed, Scopus, Web of Science) and grey literature will be searched for eligible papers. We provide a narrative description of the results based on a thematic analysis. In the second stage, out of papers found in the scoping review which discuss involvement and engagement strategies, we will conduct a systematic review following PRISMA guidelines, exploring the delivery and effectiveness of these strategies. We will conduct a qualitative synthesis (Thomas and Harden, 2008). Relevant results from the quantitative studies will be extracted and placed under qualitative themes. Individual studies will be appraised through MMAT (Hong et al., 2018), we will then assess the overall confidence in each finding through GRADE-CERQual (Lewin et al., 2015). Results will be reported in a thematic and narrative way.

Ethics and dissemination: This protocol sets out how the review will be conducted to ensure rigour and transparency. Public advisors were involved in its development. Review findings will be presented at conferences and published in peer-reviewed journals.

Keywords: Big data, PPI, public involvement, patient engagement, consumer participation, governance

Strengths and limitations of this study

- This is the first review exploring public involvement and engagement in big data research
- The search is limited to studies published in English.
- Lack of clarity and consistency with the use of the terms public involvement, engagement, or big data could impact our search results. However, we will undertake additional searching techniques to mitigate this limitation.

Introduction

What is the problem?

Over the last two decades, the ongoing digitalisation of information has allowed the creation and linkage of large, multi-source health data sets to provide novel healthcare applications. This is often called 'big data', but the concept itself is unclear and heavily debated[1]. However, this growing area of research has the following characteristics: large volume, high velocity, huge variety, veracity and value (ibid). Multiple stakeholders use big data for research; clinical management; audit; service evaluation, or statistical purposes. The UK has been a global leader in big data research. Large regional projects include the Children Growing Up in Liverpool (C-GULL)[2] and the Civic Data Cooperative[3] (to name a few). The overriding aims of such initiatives are to deliver more efficient healthcare,[4] and to reduce health inequalities.[5]

For peer review only - http://bmjopen.bmj.com/site/about/guidelines.xhtml

The use of big data for research presents ethical challenges.[6] Traditionally, a person consents to participate in a research study, whereas when large quantities of data are collected, it is not often apparent how it will be (re)used in the future. Initially, data can be collected for one purpose (e.g. audit or to collect groups statistics) and only later shared or linked for research. Secondly, even when big data is anonymised, in theory, individuals can be still re-identified.[6] Thirdly, digitalised data needs to be stored- sometimes in various places and hosted by both public institutions and private companies. Despite these ethical issues, the literature shows that the public mostly supports big data usage in research,[7] but is sceptical toward current governance mechanisms[8] and concerned about associated risks such as breach of privacy, generating waste of unused information and usage of data for profit rather than a public good. Big data is still new, and thus it often outpaces governance structures and regulation. Even if researchers meet the legal requirements, the public might not be supportive of their actions.[9 10]Controversial cases can undermine public trust in big data. For example, the case of DeepMind in the UK illustrated these dangers: the NHS breached data protection legislation by sharing patients' data (without properly informing them) with the Google-owned company.[11] Low public engagement and lack of transparency in care.data project in the UK[12] led to its eventual closure. The public might perceive the risk-benefit ratio as unfavourable for them and not want to support or participate in the research. Also, it could foster general distrust in healthcare professionals.

What is the solution?

The concept of trust is vital in building a positive relationship between researchers and the public[8]. Improving people's knowledge, through public engagement, of how big data research works can improve public support for using health data.[13] For example, #DataSavesLives initiative raises awareness of health data research benefits to gain public trust[14]. Secondly, researchers should involve the public in developing transparent, accountable policies and governance processes.[15] Public involvement and engagement are crucial mechanisms to develop governance policies and build trust between the public and researchers. Public involvement should be genuine. It should not be carried out with the sole aim of benefiting researchers; be tokenistic or mislead the public.[16] Extensive evidence shows that successful public involvement can lead to service improvement,[17-20] raises awareness of services,[20] and brings together patients' and researchers' priorities.[21]

Public involvement in big data research has context-related challenges. In traditional research, a participant and a researcher would have some contact. In contrast, big data research includes large groups of people (who might not necessarily be aware that a particular research team uses their data), thus creating a feeling of remoteness between researchers and the public.[10] Therefore, building trust between the public and researchers is more challenging. Transparent governance policies need to be developed with public involvement to ensure transparency. Lay people can be members of ethics and governance committees overseeing research projects, ensuring public voices are heard. However, the literature on public involvement and engagement in big data research is still limited. Researchers need big data specific recommendations on involving and engaging the public, which are not available to them.

Why is this review needed?

Systematic and narrative reviews that have explored the public and big data have typically focused on trust or public attitudes towards using big data for research.[7 22-24] However, how and to what extent public involvement and engagement is used in establishing trust for big data research (e.g., organising and maintaining large health data sets and its governance policies) has received less attention. To our knowledge, there is no review covering our objectives published or registered on Prospero or Cochrane.

For peer review only - http://bmjopen.bmj.com/site/about/guidelines.xhtml

Figure 1 Here

Graphic presentation (such as logic models) are utilised in reviews to identify relevant elements and the relationships between them, guiding the parameters of the review. To better understand the complexity of public involvement and engagement in big data research, we developed a system logic model (see Figure 1) following Rohwer et al. guidance.25 This model is based on team discussion, a preliminary scoping of literature, and public advisors' feedback. We used asterixis (*) to record those sections which were suggested by public advisors. Our model puts special emphasis on four related sections: context, design of public involvement and engagement strategies, targeted population, and outcomes. As our review progresses, we will develop the logic model, and present the final version in the report of our review's findings. We hope that the model will assist in interpreting the findings and identifying gaps in the literature.

Review objectives

The purpose of this review is to synthesise the evidence around public involvement and engagement in big data research. We have two objectives:

1. Comprehensively map current evidence on public involvement and engagement in big data research (scoping review).
2. Utilise this to synthesise evidence on the delivery and effectiveness of involvement and engagement strategies (systematic review).

Methods and analysis

Design

The review will be conducted in two stages as illustrated in Figure 2.[26] Firstly, the literature on public involvement and engagement in big data research will be explored by conducting a scoping review. We follow Arksey and O'Malley[27] framework and its further iterations.[28 29] The scoping review allows us to clarify concepts, illustrate current evidence in the field and gaps in research. In the second stage, out of papers identified in the scoping review, we will extract those discussing involvement and engagement strategies to explore their delivery and effectiveness. The findings from the systematic review will inform researchers on best practice and identify any conflicting views. To further enhance the quality of this review, we follow PRISMA reporting guidelines.[30]

Figure 2 here

Stage 1: Scoping review

Search strategy

We will search the following databases CINAHL, Health Research Premium Collection, PubMed, Scopus, Web of Science and check sources of grey literature related to public involvement such as the Patient-Centred Outcome Research Institute and the INVOLVE library of research projects. The first hundred hits (to be inclusive but practical) of Google Scholar search results will be scanned for inclusion. We will also hand-search papers in the journals Health Expectations, BMC Research Involvement and Engagement, and International Journal of Population Data Science. This will be followed by snowball sampling where we check references in included papers to identify additional studies for inclusion and consult with experts about other papers. Big data research is a newly developing field; for instance, MeSH terms 'big data' was added in 2019. We will restrict searches to a start date of 2010 and will update our searches prior to the final submission of our findings.

For peer review only - http://bmjopen.bmj.com/site/about/guidelines.xhtml

We developed the search strategy in partnership with an information specialist and tested this through an iterative process. It consists of both Boolean operators and where possible MeSH (PubMed) or subject heading (CINAHL). Three databases that were searched in a test run yielded a large number of references that were not relevant to our review aims. Therefore, we decided to include the further term "data governance" as we expect that most of the public involvement and engagement in big data research would be at the stage of developing and maintaining data sets. The summary of the search strategy is presented in Table 1.

Public	"advisory group" OR carer* OR citizen* OR client* OR communit* OR consumer* OR famil* OR lay OR nonpatient* OR participant* OR patient* OR public OR relative* OR representative* OR stakeholder* OR survivor* OR user*
Involvement or engagement	advocacy OR collaborat* OR co*production OR consult* OR empower* OR engage* evaluat* OR involv* OR particip* OR partner* OR PPI OR organi*ation* OR representation*
Big data	"big data" OR "data science" OR "data mining" OR "datasets" OR "data analytics" OR "data sets"
Public Involvement	"patient participation" OR "consumer participation" OR "client participation" OR "community participation"
Data governance (only Health Research Premium Collection, Scopus & Web of Science)	"data governance"

Table 1 Search strategy

Inclusion & exclusion criteria in the scoping review

Public involvement and engagement can take place at any stage of a big data research project. Thus, we include papers relating to any public role or contribution to big data research. These roles can include permission to use data, involvement in defining aims or design, and participation in decision-making processes (also the public may become members of a research team).[16]

Previous reviews[17 31 32] have noted that a lack of one generally accepted definition of public involvement makes searching databases challenging. Hence, the definition of public involvement and engagement in the literature lacks consistency.[20] Involvement, engagement, participation are often used interchangeably in the literature but do not necessarily have the same meaning.[33] We follow the INVOLVE definition for public involvement and engagment[34]:

Public involvement – "*research being carried out 'with' or 'by' members of the public rather than 'to', 'about' or 'for' them.*"

Consultation – researchers discussed the project with members of the public. It was more of 'to', 'about' or 'for' rather than 'with' or 'by' them.

Public engagement – "*information and knowledge about research is provided and disseminated.*" – this usually takes place after the project is concluded.

For peer review only - http://bmjopen.bmj.com/site/about/guidelines.xhtml

For peer review only

INVOLVE's definition of involvement sees an equal relationship between researchers and the public. Thus, involvement should mean co-design and co-production rather than just consultation. However, we will not exclude papers that do not meet this requirement but note it. Thus, included papers will be assigned one of three named categories: green (when it meets the definition of public involvement), blue (when consultation took place) and amber (where only the engagement occurred).

Multiple definitions of big data exist.[1] To broadly map the current evidence, we use a definition which focuses on big data in the healthcare setting:

Big data – data which is challenging to manage through traditional analytic tools and meets the 5V characteristics: volume, velocity, variety, veracity and value.[1]

The volume suggests that there may be a high quantity of data available potentially on millions of patients. The variety means heterogeneity of data collected as it can come in various formats (e.g. images, text). The velocity means that it can be collected swiftly from various sources. The veracity calls for accuracy and identification of any biases. The value refers to the ability of results from research based on big data to guide decisions. Big data sources can be internal (e.g. patients record, healthcare professional notes, generated through apps or social media) and external (e.g. private companies or governmental institutions).

To map a range of studies, we will keep the selection criteria purposefully broad. Papers can discuss single research projects or data sharing initiatives. All study designs will be included. Papers can be (but not limited to) original research, an evaluation, a review, an expert opinion, or a commentary that explores any public involvement and engagement aspects in big data research.

We will exclude a paper if it:
- does not discuss public involvement or engagement
- does not discuss a patient-related (or health-related) application
- the full text is not available in English

Study selection

We will scan a paper's eligibility, based on the title and then the abstract identified in the database searches. At each stage, two reviewers will be involved. The first reviewer will scan all papers and the second will check the random sample (20% of all papers). Reasons for exclusion will be recorded. If there are any disagreements, we will include a third reviewer. Then the full text will be screened, checking if the paper meets inclusion and exclusion criteria.

Data extraction

We will use an extraction form which will cover the following information:

- Paper aim
- Design
- Country
- Demographics of participants (also record if there are a seldom-heard group)
- Context
- Process of involvement or engagement
- Funding
- Legal or ethical issues
- References to guidance & policies
- Challenges and facilitators of public involvement and engagement

For peer review only - http://bmjopen.bmj.com/site/about/guidelines.xhtml

We see the extraction stage as an iterative process. After extracting initial papers, we will discuss if the extraction form is applicable in our review during team meetings. Where necessary, we will revise it.

Reporting the results

We will provide a descriptive and narrative analysis of the data. These will be used to develop the system model. Then, we discuss what are the implication of the findings for researchers and policy.

Stage 2: Systematic review

Criteria for inclusion

Out of papers identified in the scoping review, we will extract qualitative and quantitative studies that discuss the delivery or effectiveness of involvement and engagement strategies.

Data extraction and synthesis

We follow Thomas and Harden[35] stages of qualitative synthesis. We extract all findings sections from included papers and upload them to NVivo for analysis. Coding will be done inductively to develop descriptive themes to further our review aims and develop the system model. Thus, we want to ensure that no prior framework will influence us in identifying the relevant evidence. The quantitative studies' relevant results will be extracted and placed under the qualitative themes as we do not expect that meta-analysis will be possible. At the last stage of the synthesis, we go beyond the descriptive themes and analyse them in the context of our review aim. The results will be provided in a thematic, narrative way and utilised to develop the system model.

Studies and Findings Appraisal

Using MMAT[36] we will systematically appraise all studies included in the systematic review. However, no paper will be excluded if it scored low. The overall confidence in each individual qualitative finding will be assessed through GRADE-CERQual.[37] We will not assess the overall confidence in quantitative studies as these will be placed under the qualitative themes. This will allow researchers and healthcare professional to make judgments about the quality of available evidence.

Patient and public involvement

Stakeholders (including patients and health professionals) can be involved in systematic reviews.[38 39] They can enhance the quality of the review by advising on the review questions and its scope. This ensures transparency and accountability, especially if the review aims to shape practice and improves relevance to those who this review seeks to influence (e.g. practitioners and public). Similarly, for scoping reviews. Arksey[27] recommends, and Levac et al.[29] argue that consultation is a part of the review process. We have involved two public advisors who assisted in designing this protocol and will be co-authors on all publications. They will contribute throughout the review process with a particular emphasis on interpreting the findings and developing further recommendations for both research and practice.

Limitations

The main limitation of our review is the exclusion of non-English papers. There is a possibility that some papers relevant to our review aims will be excluded and this will impact our findings. Secondly, as already mentioned the lack of clear definitions of public involvement. engagement and big data make any search strategy challenging, and potentially some

For peer review only - http://bmjopen.bmj.com/site/about/guidelines.xhtml

relevant papers might be not be included. However, we will undertake all reasonable steps to balance this limitation by reaching experts and checking references in included papers.

Ethics and dissemination

We publish this protocol and engaged with public advisors to ensure transparency and rigour of our review process. As we use already published data, there is no need to apply for ethical approval to conduct our study. We will present our findings at relevant conferences and publish in a peer-reviewed journal.

Conclusions

This review will synthesise the current literature on public involvement and engagement in big data research. Our work is timely as it is expected that big data research in healthcare will continue to grow rapidly. There will be increasing interest in developing large health data sets by researchers, funders, and governmental bodies. Previous research shows the need for synthesising the current evidence. Mouton et al[40] discussed issues around patient trust and big data, and how they viewed healthcare practitioners and professionals' involvement in funding or controlling big data research. They believed that patients were not interested or understood big data– and therefore, should not be involved in its governance. Their comments included remarks that the patients' groups lack relevance and the belief that patients' involvement in governance would be pointless. On the other hand, Aitken, et al. [8] explored the similar issues with members of the public who presented opposite views on lay involvement in data governance. Participants believed that members of the public could promote accountability of big data research. Public involvement has the potential to shift perspectives and bridge the gap between researchers and the public, and help the development of big data research that has wider spread public support and buy-in.

For peer review only

Authors contributions: PT developed the study design, drafted the protocol and conducted initial searches with the assistance of the librarian. EJ, NT, SA, LF contributed to drafting and editing. All authors have read and approved the final manuscript.

Acknowledgement: The development of the search strategy was supported by Zoe Gibbs-Monaghan Liaison Librarian – Health and Life Sciences at the University of Liverpool. Ruaraidh Hill, Lecturer in evidence synthesis at the University of Liverpool provided beneficial insights into review's methodology.

Funding statement: Piotr Teodorowski is a PhD student supported by the National Institute for Health Research Applied Research Collaboration North West Coast (NIHR ARC NWC) Award number: 103. The views expressed in this publication are those of the author(s) and not necessarily those of the National Institute for Health Research or the Department of Health and Social Care.

Data statement: Data sharing not applicable.

Competing interests: None declared

For peer review only

For peer review only - http://bmjopen.bmj.com/site/about/guidelines.xhtml

References

1. Mehta N, Pandit A. Concurrence of big data analytics and healthcare: A systematic review. *International Journal of Medical Informatics* 2018;114:57-65. doi: 10.1016/j.ijmedinf.2018.03.013
2. University of Liverpool. Children Growing Up in Liverpool (C-GULL), 2020.
3. LCR. Civic Data Co-operative 2020 [Available from: https://www.liverpoolcityregion-ca.gov.uk/liverpool-city-region-combined-authority-announces-proposals-for-5-3m-funding-for-data-driven-health-improvements/.
4. Raghupathi W, Raghupathi V. Big data analytics in healthcare: promise and potential. *Health Information Science and Systems* 2014;2(1):3. doi: 10.1186/2047-2501-2-3
5. Zhang X, Pérez-Stable EJ, Bourne PE, et al. Big Data Science: Opportunities and Challenges to Address Minority Health and Health Disparities in the 21st Century. *Ethnicity & Disease* 2017;27(2):95. doi: 10.18865/ed.27.2.95
6. Lipworth W, Mason PH, Kerridge I, et al. Ethics and Epistemology in Big Data Research. *Journal of Bioethical Inquiry* 2017;14(4):489-500. doi: 10.1007/s11673-017-9771-3
7. Aitken M, de St. Jorre J, Pagliari C, et al. Public responses to the sharing and linkage of health data for research purposes: a systematic review and thematic synthesis of qualitative studies. *BMC Medical Ethics* 2016;17(1):73. doi: 10.1186/s12910-016-0153-x
8. Aitken M, Cunningham-Burley S, Pagliari C. Moving from trust to trustworthiness: Experiences of public engagement in the Scottish Health Informatics Programme. *Science and Public Policy* 2016;43(5):713-23. doi: 10.1093/scipol/scv075
9. Carter P, Laurie GT, Dixon-Woods M. The social licence for research: why care.data ran into trouble. *Journal of Medical Ethics* 2015;41(5):404. doi: 10.1136/medethics-2014-102374
10. Aitken M, Tully M, Porteous C, et al. Consensus Statement on Public Involvement and Engagement with Data-Intensive Health Research. *International Journal of Population Data Science* 2019;4 doi: 10.23889/ijpds.v4i1.586
11. Revell T. Google DeepMind's NHS data deal 'failed to comply' with law New Scientist2017 [Available from: https://institutions.newscientist.com/article/2139395-google-deepminds-nhs-data-deal-failed-to-comply-with-law/.
12. Taylor M. Information Governance as a Force for Good? Lessons to be Learnt from Care.data. *SCRIPTed* 2014;11 doi: 10.2966/scrip.110114.1
13. Hill EM, Turner EL, Martin RM, et al. "Let's get the best quality research we can": public awareness and acceptance of consent to use existing data in health research: a systematic review and qualitative study. *BMC Medical Research Methodology* 2013;13(1):72. doi: 10.1186/1471-2288-13-72
14. Data Saves Lives. What is Data Saves Lives? 2020 [Available from: https://datasaveslives.eu/aboutdsl accessed 11 January 2021.
15. Ford E, Boyd A, Bowles JKF, et al. Our data, our society, our health: A vision for inclusive and transparent health data science in the United Kingdom and beyond. *Learning Health Systems* 2019;3(3):e10191. doi: 10.1002/lrh2.10191
16. Beier K, Schweda M, Schicktanz S. Taking patient involvement seriously: a critical ethical analysis of participatory approaches in data-intensive medical research. *BMC Medical Informatics and Decision Making* 2019;19(1) doi: 10.1186/s12911-019-0799-7
17. Lalani M, Baines R, Bryce M, et al. Patient and public involvement in medical performance processes: A systematic review. *Health expectations : an international journal of public participation in health care and health policy* 2019;22(2):149-61. doi: 10.1111/hex.12852 [published Online First: 2018/12/11]
18. Ocloo J, Matthews R. From tokenism to empowerment: progressing patient and public involvement in healthcare improvement. *BMJ Quality & Safety* 2016;25(8):626-32. doi: 10.1136/bmjqs-2015-004839

For peer review only

For peer review only - http://bmjopen.bmj.com/site/about/guidelines.xhtml

19. Crawford MJ. Systematic review of involving patients in the planning and development of health care. *BMJ* 2002;325(7375):1263-63. doi: 10.1136/bmj.325.7375.1263
20. Mockford C, Staniszewska S, Griffiths F, et al. The impact of patient and public involvement on UK NHS health care: a systematic review. *International Journal for Quality in Health Care* 2012;24(1):28-38. doi: 10.1093/intqhc/mzr066
21. Sofolahan-Oladeinde Y, Newhouse RP, Lavallee DC, et al. Early assessment of the 10-step patient engagement framework for patient-centred outcomes research studies: the first three steps. *Family Practice* 2017;34(3):272-77. doi: 10.1093/fampra/cmx013
22. Stockdale J, Cassell J, Ford E. “Giving something back”: A systematic review and ethical enquiry into public views on the use of patient data for research in the United Kingdom and the Republic of Ireland. *Wellcome Open Research* 2019;3:6. doi: 10.12688/wellcomeopenres.13531.2
23. Kalkman S, Van Delden J, Banerjee A, et al. Patients’ and public views and attitudes towards the sharing of health data for research: a narrative review of the empirical evidence. *Journal of Medical Ethics* 2019:medethics-2019-. doi: 10.1136/medethics-2019-105651
24. Howe N, Giles E, Newbury-Birch D, et al. Systematic review of participants’ attitudes towards data sharing: a thematic synthesis. *Journal of Health Services Research & Policy* 2018;23(2):123-33. doi: 10.1177/1355819617751555
25. Rohwer A, Pfadenhauer L, Burns J, et al. Series: Clinical Epidemiology in South Africa. Paper 3: Logic models help make sense of complexity in systematic reviews and health technology assessments. *Journal of Clinical Epidemiology* 2017;83:37-47. doi: 10.1016/j.jclinepi.2016.06.012
26. Gough D, Thomas J, Oliver S. Clarifying differences between review designs and methods. *Systematic Reviews* 2012;1(1):28. doi: 10.1186/2046-4053-1-28
27. Arksey H, O'Malley L. Scoping studies: towards a methodological framework. *International Journal of Social Research Methodology* 2005;8(1):19-32. doi: 10.1080/1364557032000119616
28. Colquhoun HL, Levac D, O'Brien KK, et al. Scoping reviews: time for clarity in definition, methods, and reporting. *Journal of Clinical Epidemiology* 2014;67(12):1291-94. doi: 10.1016/j.jclinepi.2014.03.013
29. Levac D, Colquhoun H, O'Brien KK. Scoping studies: advancing the methodology. *Implementation Science* 2010;5(1):69. doi: 10.1186/1748-5908-5-69
30. Moher D, Shamseer L, Clarke M, et al. Preferred reporting items for systematic review and meta-analysis protocols (PRISMA-P) 2015 statement. *Systematic Reviews* 2015;4(1):1. doi: 10.1186/2046-4053-4-1
31. Dawson S, Campbell SM, Giles SJ, et al. Black and minority ethnic group involvement in health and social care research: A systematic review. *Health Expectations* 2018;21(1):3-22. doi: 10.1111/hex.12597
32. Harrison JD, Auerbach AD, Anderson W, et al. Patient stakeholder engagement in research: A narrative review to describe foundational principles and best practice activities. *Health Expectations* 2019;22(3):307-16. doi: 10.1111/hex.12873
33. Islam S, Small N. An annotated and critical glossary of the terminology of inclusion in healthcare and health research. *Research Involvement and Engagement* 2020;6(1):14. doi: 10.1186/s40900-020-00186-6
34. INVOLVE. What is public involvement in research? 2020 [Available from: https://www.invo.org.uk/find-out-more/what-is-public-involvement-in-research-2/ accessed 21 September 2020.
35. Thomas J, Harden A. Methods for the thematic synthesis of qualitative research in systematic reviews. *BMC Medical Research Methodology* 2008;8(1):45. doi: 10.1186/1471-2288-8-45
36. Hong Q, Pluye P, Fàbregues S, et al. Mixed Methods Appraisal Tool (MMAT) 2018 [Available from:

For peer review only - http://bmjopen.bmj.com/site/about/guidelines.xhtml

http://mixedmethodsappraisaltoolpublic.pbworks.com/w/file/fetch/127916259/MMAT_2018_criteria-manual_2018-08-01_ENG.pdf accessed 12/11 2020.
37. Lewin S, Glenton C, Munthe-Kaas H, et al. Using Qualitative Evidence in Decision Making for Health and Social Interventions: An Approach to Assess Confidence in Findings from Qualitative Evidence Syntheses (GRADE-CERQual). *PLOS Medicine* 2015;12(10):e1001895. doi: 10.1371/journal.pmed.1001895
38. Cottrell EK, Whitlock EP, Kato E, et al. Defining the benefits and challenges of stakeholder engagement in systematic reviews. *Comparative Effectiveness Research* 2015;5:13-19. doi: http://dx.doi.org/10.2147/CER.S69605
39. INVOLVE. Public involvement in systematic reviews: Supplement to the briefing notes for researchers. Eastleigh: INVOLVE, 2012.
40. Mouton Dorey C, Baumann H, Biller-Andorno N. Patient data and patient rights: Swiss healthcare stakeholders' ethical awareness regarding large patient data sets - a qualitative study. *BMC medical ethics* 2018;19(1):20-20. doi: 10.1186/s12910-018-0261-x

For peer review only

For peer review only - http://bmjopen.bmj.com/site/about/guidelines.xhtml

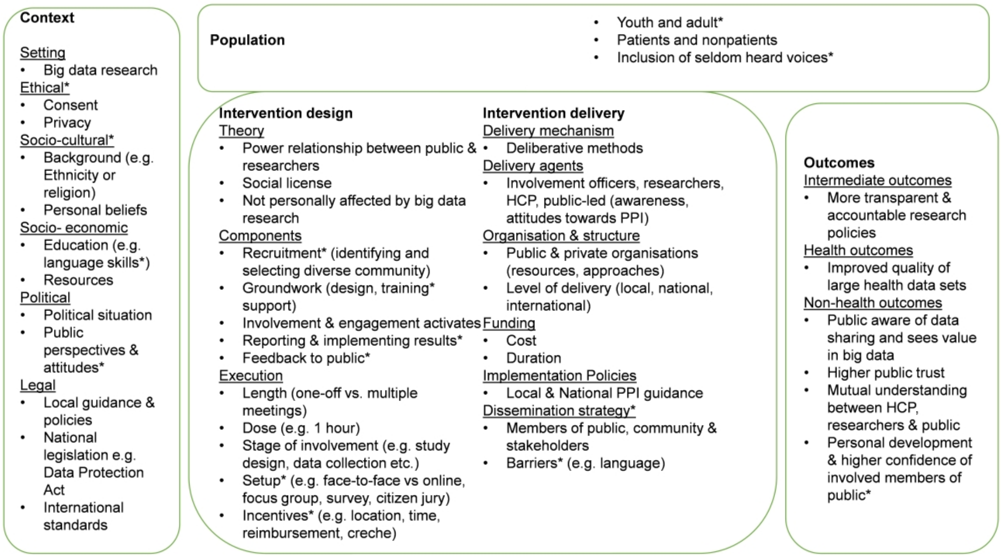

Figure 1 System logic model of public involvement and engagement in big data research.

677x381mm (300 x 300 DPI)

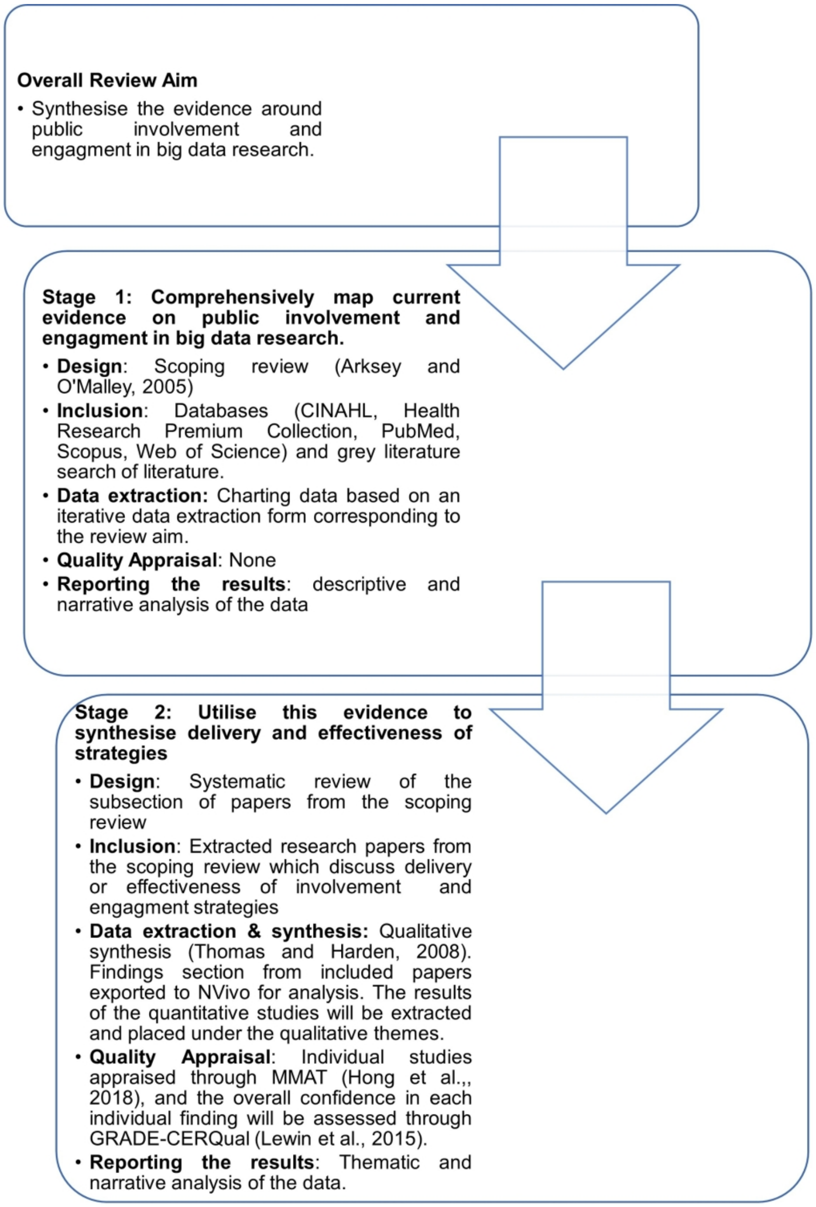

Figure 2 Systematic map of the review process

175x257mm (300 x 300 DPI)

Reporting checklist for protocol of a systematic review and meta analysis.

Based on the PRISMA-P guidelines.

Instructions to authors

Complete this checklist by entering the page numbers from your manuscript where readers will find each of the items listed below.

Your article may not currently address all the items on the checklist. Please modify your text to include the missing information. If you are certain that an item does not apply, please write "n/a" and provide a short explanation.

Upload your completed checklist as an extra file when you submit to a journal.

In your methods section, say that you used the PRISMA-Preporting guidelines, and cite them as:

Moher D, Shamseer L, Clarke M, Ghersi D, Liberati A, Petticrew M, Shekelle P, Stewart LA. Preferred Reporting Items for Systematic Review and Meta-Analysis Protocols (PRISMA-P) 2015 statement. Syst Rev. 2015;4(1):1.

		Reporting Item	Page Number
**Title**			
Identification	#1a	Identify the report as a protocol of a systematic review	2
Update	#1b	If the protocol is for an update of a previous systematic review, identify as such	n/a

For peer review only

**Registration**			
	#2	If registered, provide the name of the registry (such as PROSPERO) and registration number	n/a as this protocol includes scoping review
**Authors**			
Contact	#3a	Provide name, institutional affiliation, e-mail address of all protocol authors; provide physical mailing address of corresponding author	1
Contribution	#3b	Describe contributions of protocol authors and identify the guarantor of the review	9
**Amendments**			
	#4	If the protocol represents an amendment of a previously completed or published protocol, identify as such and list changes; otherwise, state plan for documenting important protocol amendments	n/a
**Support**			
Sources	#5a	Indicate sources of financial or other support for the review	9
Sponsor	#5b	Provide name for the review funder and / or sponsor	9

For peer review only

Role of sponsor or funder	#5c	Describe roles of funder(s), sponsor(s), and / or institution(s), if any, in developing the protocol	9
**Introduction**			
Rationale	#6	Describe the rationale for the review in the context of what is already known	2-3
Objectives	#7	Provide an explicit statement of the question(s) the review will address with reference to participants, interventions, comparators, and outcomes (PICO)	4-6
**Methods**			
Eligibility criteria	#8	Specify the study characteristics (such as PICO, study design, setting, time frame) and report characteristics (such as years considered, language, publication status) to be used as criteria for eligibility for the review	5-6 & 7
Information sources	#9	Describe all intended information sources (such as electronic databases, contact with study authors, trial registers or other grey literature sources) with planned dates of coverage	5
Search strategy	#10	Present draft of search strategy to be used for at least one electronic database, including planned limits, such that it could be repeated	5
Study records - data management	#11a	Describe the mechanism(s) that will be used to manage records and data throughout the review	6

For peer review only

Study records - selection process	#11b	State the process that will be used for selecting studies (such as two independent reviewers) through each phase of the review (that is, screening, eligibility and inclusion in meta-analysis)	6-7
Study records - data collection process	#11c	Describe planned method of extracting data from reports (such as piloting forms, done independently, in duplicate), any processes for obtaining and confirming data from investigators	6
Data items	#12	List and define all variables for which data will be sought (such as PICO items, funding sources), any pre-planned data assumptions and simplifications	n/a
Outcomes and prioritization	#13	List and define all outcomes for which data will be sought, including prioritization of main and additional outcomes, with rationale	n/a
Risk of bias in individual studies	#14	Describe anticipated methods for assessing risk of bias of individual studies, including whether this will be done at the outcome or study level, or both; state how this information will be used in data synthesis	7
Data synthesis	#15a	Describe criteria under which study data will be quantitatively synthesised	n/a
Data synthesis	#15b	If data are appropriate for quantitative synthesis, describe planned summary measures, methods of handling data and methods of combining data from	n/a

For peer review only

		studies, including any planned exploration of consistency (such as I2, Kendall's τ)	
Data synthesis	#15c	Describe any proposed additional analyses (such as sensitivity or subgroup analyses, meta-regression)	n/a
Data synthesis	#15d	If quantitative synthesis is not appropriate, describe the type of summary planned	7
Meta-bias(es)	#16	Specify any planned assessment of meta-bias(es) (such as publication bias across studies, selective reporting within studies)	n/a
Confidence in cumulative evidence	#17	Describe how the strength of the body of evidence will be assessed (such as GRADE)	7

None The PRISMA-P elaboration and explanation paper is distributed under the terms of the Creative Commons Attribution License CC-BY. This checklist can be completed online using https://www.goodreports.org/, a tool made by the EQUATOR Network in collaboration with Penelope.ai

For peer review only

BMJ Open

Public involvement and engagement in big data research: protocol for a scoping review and a systematic review of delivery and effectiveness of strategies for involvement and engagement.

Journal:	*BMJ Open*
Manuscript ID	bmjopen-2021-050167.R1
Article Type:	Protocol
Date Submitted by the Author:	30-Jun-2021
Complete List of Authors:	Teodorowski, Piotr; University of Liverpool, Institute of Population Health<br>Jones, Elisa; University of Liverpool, Institute of Population Health<br>Tahir, Naheed; ARC NWC Public Advisor<br>Ahmed, Saiqa; ARC NWC Public Advisor<br>Frith, Lucy; University of Liverpool, Departments of Law and Philosophy
**Primary Subject Heading**:	Ethics
Secondary Subject Heading:	Ethics
Keywords:	PUBLIC HEALTH, QUALITATIVE RESEARCH, MEDICAL ETHICS

SCHOLARONE™
Manuscripts

I, the Submitting Author has the right to grant and does grant on behalf of all authors of the Work (as defined in the below author licence), an exclusive licence and/or a non-exclusive licence for contributions from authors who are: i) UK Crown employees; ii) where BMJ has agreed a CC-BY licence shall apply, and/or iii) in accordance with the terms applicable for US Federal Government officers or employees acting as part of their official duties; on a worldwide, perpetual, irrevocable, royalty-free basis to BMJ Publishing Group Ltd ("BMJ") its licensees and where the relevant Journal is co-owned by BMJ to the co-owners of the Journal, to publish the Work in this journal and any other BMJ products and to exploit all rights, as set out in our licence.

The Submitting Author accepts and understands that any supply made under these terms is made by BMJ to the Submitting Author unless you are acting as an employee on behalf of your employer or a postgraduate student of an affiliated institution which is paying any applicable article publishing charge ("APC") for Open Access articles. Where the Submitting Author wishes to make the Work available on an Open Access basis (and intends to pay the relevant APC), the terms of reuse of such Open Access shall be governed by a Creative Commons licence – details of these licences and which Creative Commons licence will apply to this Work are set out in our licence referred to above.

Other than as permitted in any relevant BMJ Author's Self Archiving Policies, I confirm this Work has not been accepted for publication elsewhere, is not being considered for publication elsewhere and does not duplicate material already published. I confirm all authors consent to publication of this Work and authorise the granting of this licence.

For peer review only

Title: Public involvement and engagement in big data research: protocol for a scoping review and a systematic review of delivery and effectiveness of strategies for involvement and engagement.

Authors:

Piotr Teodorowski, Institute of Population Health, University of Liverpool, p.teodorowski@liverpool.ac.uk ORCID: 0000-0003-2172-8298

Elisa Jones, Institute of Population Health, University of Liverpool, elisa.jones@liverpool.ac.uk

Naheed Tahir, ARC NWC Public Adviser, naheedtahir33@hotmail.co.uk

Saiqa Ahmed, ARC NWC Public Adviser, saiqa75@hotmail.co.uk

Lucy Frith, Departments of Law and Philosophy, University of Liverpool, frith@liverpool.ac.uk, ORCID: 0000-0002-8506-0699

Corresponding author: Piotr Teodorowski

Word count: 2880

For peer review only - http://bmjopen.bmj.com/site/about/guidelines.xhtml

Public involvement and engagement in big data research: protocol for a scoping review and a systematic review of delivery and effectiveness of strategies for involvement and engagement.

Abstract

Introduction: Big data research has grown considerably over the last two decades. This presents new ethical challenges around consent, data storage and anonymisation. Big data research projects require public support to succeed and it has been argued that one way to achieve this is through public involvement and engagement. To better understand the role public involvement and engagement can play in big data research, we will review the current literature. This protocol describes the planned review methods.

Methods and analysis: Our review will be conducted in two stages. In the first stage, we will conduct a scoping review using Arksey and O'Malley (2005) methodology to comprehensively map current evidence on public involvement and engagement in big data research. Databases (CINAHL, Health Research Premium Collection, PubMed, Scopus, Web of Science) and grey literature will be searched for eligible papers. We provide a narrative description of the results based on a thematic analysis. In the second stage, out of papers found in the scoping review which discuss involvement and engagement strategies, we will conduct a systematic review following PRISMA guidelines, exploring the delivery and effectiveness of these strategies. We will conduct a qualitative synthesis (Thomas and Harden, 2008). Relevant results from the quantitative studies will be extracted and placed under qualitative themes. Individual studies will be appraised through MMAT (Hong et al., 2018), we will then assess the overall confidence in each finding through GRADE-CERQual (Lewin et al., 2015). Results will be reported in a thematic and narrative way.

Ethics and dissemination: This protocol sets out how the review will be conducted to ensure rigour and transparency. Public advisors were involved in its development. Ethics approval is not required. Review findings will be presented at conferences and published in peer-reviewed journals.

Keywords: Big data, PPI, public involvement, patient engagement, consumer participation, governance

Strengths and limitations of this study

- This is the first review exploring public involvement and engagement in big data research
- The search is limited to studies published in English.
- Lack of clarity and consistency with the use of the terms public involvement, engagement, and big data could impact our search results. However, we will undertake additional searching techniques to mitigate this limitation.

Introduction

What is the problem?

Over the last two decades, the ongoing digitalisation of information has allowed the creation and linkage of large, multi-source health data sets to provide novel healthcare applications. This is often called ‘big data’, but the concept itself is unclear and heavily debated[1]. However, this growing area of research has the following characteristics: large volume, high velocity, huge variety, veracity and value (ibid). Multiple stakeholders use big data for research; clinical management; audit; service evaluation, or statistical purposes. The UK has been a global leader in big data research. Large projects include, at national level, OpenSAFELY[2] and regionally located projects such as Children Growing Up in Liverpool (C-GULL)[3] (to name a few). The overriding aims of big data research projects are to deliver more efficient healthcare,[4] and to reduce health inequalities.[5]

For peer review only - http://bmjopen.bmj.com/site/about/guidelines.xhtml

The use of big data for research presents ethical challenges.[6] Traditionally, a person consents to participate in a research study, whereas when large quantities of data are collected, it is not often apparent how it will be (re)used in the future. Data can be collected for one purpose (e.g. audit or to collect groups statistics) and only later shared or linked for research. Secondly, even when big data is anonymised, in theory, individuals can be still re-identified.[6] Thirdly, digitalised data needs to be stored - sometimes in various places and hosted by both public institutions and private companies. Despite these ethical issues (consent, anonymisation, data storage and access), the literature shows that the public mostly supports big data usage in research,[7] but is sceptical toward current governance mechanisms[8] and concerned about associated risks such as breach of privacy, generating waste of unused information and usage of data for profit rather than for the public good. Big data is still new, and thus it often outpaces governance structures and regulation. Even if researchers meet the legal requirements, the public might not be supportive of their actions.[9 10]Controversial cases can undermine public trust in big data. For example, the case of DeepMind in the UK illustrated these dangers: the NHS breached data protection legislation by sharing patients' data (without properly informing them) with the Google-owned company.[11] Low public engagement and lack of transparency in the care.data project in the UK[12] led to its eventual closure. The public might perceive the risk-benefit ratio as unfavourable for them and therefore not want to support or participate in the research. Also, it could foster general distrust in healthcare professionals.

What is the solution?

The concept of trust is vital in building a positive relationship between researchers and the public[8]. Improving people's knowledge, through public engagement, of how big data research works can improve public support for using health data.[13] For example, the #DataSavesLives initiative raises awareness of the benefits of health data research to gain public trust[14]. Secondly, researchers should involve the public in developing transparent, accountable policies and governance processes.[15] Public involvement and engagement are crucial mechanisms to develop governance policies and build trust between the public and researchers. Public involvement should be genuine. It should not be carried out with the sole aim of benefiting researchers; be tokenistic or mislead the public.[16] Extensive evidence shows that successful public involvement can lead to service improvement,[17-20] raises awareness of services,[20] and brings together patients' and researchers' priorities.[21]

Public involvement in big data research has context-related challenges. In traditional research, a participant and a researcher would have some contact. In contrast, big data research includes large groups of people (who might not necessarily be aware that a particular research team uses their data), thus creating a feeling of remoteness between researchers and the public.[10] Therefore, building trust between the public and researchers is more challenging. Transparent governance policies need to be developed with public involvement to ensure transparency. Lay people can be members of ethics and governance committees overseeing research projects, ensuring public voices are heard. Researchers need big data specific recommendations on involving and engaging the public. However, the literature on public involvement and engagement in big data research is still limited.

Why is this review needed?

Systematic and narrative reviews that have explored the public attitudes towards big data have typically focused on trust or attitudes towards using big data for research.[7 22-24] However, how and to what extent public involvement and engagement is used in establishing trust for big data research (e.g., organising and maintaining large health data sets and its governance policies) has received less attention. To our knowledge, there is no review covering our objectives published or registered on Prospero or Cochrane databases.

For peer review only - http://bmjopen.bmj.com/site/about/guidelines.xhtml

Figure 1 Here

To better understand the complexity of public involvement and engagement in big data research, we developed a system logic model (see Figure 1) following Rohwer et al. guidance.[25] Graphic presentations (such as logic models) can be used in reviews to identify relevant elements and the relationships between them. This model is based on team discussion, a preliminary scoping of literature, and public advisors' feedback. We used asterixis (*) to record those sections which were suggested by public advisors. Our model puts special emphasis on four related sections: context, design of public involvement and engagement strategies, targeted population, and outcomes. As our review progresses, we will develop the logic model, and present the final version in the report of our review's findings. We hope that the model will assist in interpreting the findings and identifying gaps in the literature.

Review objectives

The purpose of this review is to synthesise the evidence on public involvement and engagement in big data research. We have two objectives:

1. Comprehensively map current evidence on public involvement and engagement in big data research (scoping review).
2. Utilise this to synthesise evidence on the delivery and effectiveness of involvement and engagement strategies (systematic review).

Methods and analysis

Design

The review will be conducted in two stages as illustrated in Figure 2.[26] These stages will complement each other and assist in flexibly understanding the phenomenon. Firstly, the literature on public involvement and engagement in big data research will be explored by conducting a scoping review. We follow Arksey and O'Malley[27] framework and its further iterations.[28 29] The scoping review will allow us to clarify concepts, illustrate current evidence in the field and gaps in research.[30] In the second stage, out of papers identified in the scoping review, we will extract those discussing involvement and engagement strategies to explore their delivery and effectiveness. The findings from the systematic review will inform researchers on best practice and identify any conflicting views.[30].To further enhance the quality of this review, we follow PRISMA reporting guidelines.[31]

Figure 2 here

Stage 1: Scoping review
Search strategy

We will search the following databases CINAHL, Health Research Premium Collection, PubMed, Scopus, Web of Science and check sources of grey literature related to public involvement such as the Patient-Centred Outcome Research Institute. The first hundred hits (to be inclusive but practical) of Google Scholar search results will be scanned for inclusion. We will also hand-search papers in the journals Health Expectations, BMC Research Involvement and Engagement, and the International Journal of Population Data Science. This will be followed by snowball sampling where we will check references in included papers to identify additional studies for inclusion and consult with experts about relevant papers. Big data research is a newly developing field; for instance, MeSH terms 'big data' was added in 2019. Thus, to capture these recent developments, we will restrict searches to a start date of 2010 and will update our searches prior to the final submission of our findings.

For peer review only - http://bmjopen.bmj.com/site/about/guidelines.xhtml

We developed the search strategy in partnership with an information specialist and tested this through an iterative process. It consists of both Boolean operators and where possible MeSH (PubMed) or subject heading (CINAHL). Three databases were searched in a test run and yielded a large number of references that were not relevant to our review aims. Therefore, we decided to include the further term "data governance" as we expect that most of the public involvement and engagement in big data research would be at the stage of developing and maintaining data sets. The summary of the search strategy is presented in Table 1.

Public	"advisory group" OR carer* OR citizen* OR client* OR communit* OR consumer* OR famil* OR lay OR nonpatient* OR participant* OR patient* OR public OR relative* OR representative* OR stakeholder* OR "steering group*" OR survivor* OR user*
Involvement or engagement	advocacy OR collaborat* OR co*production OR consult* OR empower* OR engage* evaluat* OR involv* OR particip* OR partner* OR PPI OR organi*ation* OR representation*
Big data	database OR "big data" OR "data science" OR "data mining" OR "datasets" OR "data analytics" OR "data sets"
Public Involvement	"patient participation" OR "consumer participation" OR "client participation" OR "community participation"
Data governance (only Health Research Premium Collection, Scopus & Web of Science)	"data governance"

Table 1 Search strategy

Inclusion & exclusion criteria in the scoping review

Public involvement and engagement can take place at any stage of a big data research project. Thus, we will include papers relating to any public role or contribution to big data research. These roles can include permission to use data, involvement in defining aims or design, and participation in decision-making processes (also the public may become members of a research team).[16]

Previous reviews[17 32 33] have noted that a lack of one generally accepted definition of public involvement makes searching databases challenging. Hence, the definition of public involvement and engagement in the literature lacks consistency.[20] Involvement, engagement, participation are often used interchangeably in the literature but do not necessarily have the same meaning.[34] We follow the INVOLVE definition of public involvement and engagment[35]:

Public involvement – "*research being carried out 'with' or 'by' members of the public rather than 'to', 'about' or 'for' them.*"

Consultation – researchers discussed the project with members of the public. It was more of 'to', 'about' or 'for' rather than 'with' or 'by' them.

For peer review only - http://bmjopen.bmj.com/site/about/guidelines.xhtml

Public engagement – "*information and knowledge about research is provided and disseminated.*" – this usually takes place after the project is concluded.

INVOLVE's definition of involvement sees an equal relationship between researchers and the public. Thus, involvement should mean co-design and co-production rather than just consultation. However, we will not exclude papers that do not meet this requirement but note it. Thus, included papers will be assigned one of three named categories: green (when it meets the definition of public involvement), blue (when consultation took place) and amber (where only the engagement occurred).

Multiple definitions of big data exist.[1] To broadly map the current evidence, we use a definition which focuses on big data in the healthcare setting:

Big data – data which is challenging to manage through traditional analytic tools and meets the 5V characteristics: volume, velocity, variety, veracity and value.[1]

The volume suggests that there may be a high quantity of data available potentially on millions of patients. The variety means heterogeneity of data collected as it can come in various formats (e.g. images, text). The velocity means that it can be collected swiftly from various sources. Veracity relates to the accuracy and identification of any biases. The value refers to the ability of results from research based on big data to guide decisions. Big data sources can be internal (e.g. patients record, healthcare professional notes, generated through apps or social media) and external (e.g. private companies or governmental institutions).

To map a range of studies, we will keep the selection criteria purposefully broad. Papers can discuss single research project or data sharing initiative. All study designs will be included. Papers can be (but not limited to) original research, an evaluation, a review, an expert opinion, or a commentary that explores any public involvement and engagement in big data research.

We will exclude a paper if it:

- does not discuss public involvement or engagement
- does not discuss a patient-related (or health-related) application
- the full text is not available in English

Study selection

Prior to the screening stage, we will organise a meeting for everyone involved in study selection process during which we will jointly scan a sample of 100 papers. We will record and discuss our disagreements. Then separately, we will scan all papers' eligibility, based on the title and then the abstract identified in the database searches. At each stage, two reviewers will be involved. The first reviewer will scan all papers and the second will check a random sample (20% of all papers). Reasons for exclusion will be recorded. If there are any disagreements, we will include a third reviewer. Then the full text will be screened by two reviewers, checking if the paper meets the inclusion and exclusion criteria. We will meet after each screening stage (title, abstract and full paper) to discuss ours experiences.

Data extraction

We will use an extraction form which will cover the following information:

- Paper aim
- Design
- Country
- Demographics of participants (also record if there are a seldom-heard group)
- Context

For peer review only - http://bmjopen.bmj.com/site/about/guidelines.xhtml

- Process of involvement or engagement
- Funding
- Legal or ethical issues
- References to guidance & policies
- Challenges and facilitators of public involvement and engagement

We see the extraction stage as an iterative process. After extracting initial papers, we will discuss if the extraction form is applicable in our review during team meetings. Where necessary, we will revise it. Each paper will be extracted by one reviewer and the second will validate data extraction.

Reporting the results

We will provide a descriptive and narrative analysis of the data. These will be used to develop the system model. Then, we will discuss the implication of the findings for researchers and policy.

Stage 2: Systematic review

Criteria for inclusion

Out of papers identified in the scoping review, we will extract qualitative and quantitative studies that discuss the delivery or effectiveness of involvement and engagement strategies.

Data extraction and synthesis

We will follow Thomas and Harden[36] stages of qualitative synthesis. We plan to extract all findings sections from included papers and upload them to NVivo for analysis. Coding will be done inductively to develop descriptive themes to further our review aims and develop the system model. Thus, we want to ensure that no prior framework will influence us in identifying the relevant evidence. The relevant results from the quantitative studies will be extracted and placed under qualitative themes, as we do not expect that meta-analysis will be possible. At the last stage of the synthesis, we go beyond the descriptive themes and analyse them in the context of the aims of our review. The results will be provided in a thematic, narrative way and utilised to develop the system model.

Studies and Findings Appraisal

Using MMAT[37] we will systematically appraise all studies included in the systematic review. However, no paper will be excluded if it scored low. The overall confidence in each individual paper's qualitative findings will be assessed through GRADE-CERQual.[38] We will not assess the overall confidence in quantitative studies as these will be placed under the qualitative themes. This will allow researchers to make judgments about the quality of available evidence.

Patient and public involvement

Stakeholders (including patients and health professionals) can be involved in systematic reviews.[39 40] They can enhance the quality of the review by advising on the review questions and its scope. This ensures transparency and accountability, especially if the review aims to shape practice and improves relevance to those who this review seeks to influence (e.g. practitioners and public). Similarly, for scoping reviews. Arksey[27] recommends, and Levac et al.[29] argue that consultation is a part of the review process. We have involved two public advisors who assisted in designing this protocol and will be co-authors on all publications. They have experience of conducting systematic reviews, represent seldom-heard communities and SA is a Big Data Ambassador for Care and Health Informatics theme within ARC NWC. They will be involved in the whole review process, with a particular emphasis on

For peer review only - http://bmjopen.bmj.com/site/about/guidelines.xhtml

interpreting the findings and developing recommendations for both research and practice. We will report on public involvement using the GRIPP2 checklist.[41]

Limitations

The main limitation of our review is the exclusion of non-English papers. There is a possibility that some papers relevant to our review aims will be excluded and this will impact our findings. Secondly, as already mentioned the lack of clear definitions of public involvement. engagement and big data make any search strategy challenging, and potentially some relevant papers might not be included. However, we will undertake all reasonable steps to balance this limitation by involving experts and checking references in included papers.

Ethics and dissemination

We have published this protocol and engaged with public advisors to ensure transparency and rigour of our review process. As we are using already published data, there is no need to apply for ethical approval to conduct our study. We will present our findings at relevant conferences and publish in a peer-reviewed journal.

Discussion

This review will synthesise the current literature on public involvement and engagement in big data research. Our work is timely as it is expected that big data research in healthcare will continue to grow rapidly. There will be increasing interest in developing large health data sets by researchers, funders, and governmental bodies. Previous research shows the need for synthesising the current evidence. Mouton et al[42] discussed issues around patient trust and big data, and how they viewed healthcare practitioners and professionals' involvement in funding or controlling big data research. They believed that patients were not interested or did not understand big data – and therefore, should not be involved in its governance. Their comments included remarks that patient groups are not important and the belief that patients' involvement in governance would be pointless. On the other hand, Aitken, et al. [8] explored the similar issues with members of the public who presented opposite views on lay involvement in data governance. Participants believed that members of the public could promote accountability of big data research. Public involvement has the potential to shift perspectives and bridge the gap between researchers and the public, and help the development of big data research that has wider spread public support and buy-in.

Figure 1 System logic model of public involvement and engagement in big data research.

Figure 2 Systematic map of the review process

For peer review only - http://bmjopen.bmj.com/site/about/guidelines.xhtml

Authors contributions: PT developed the study design, drafted the protocol and conducted initial searches with the assistance of the librarian. EJ, NT, SA, LF contributed to drafting and editing. All authors have read and approved the final manuscript.

Acknowledgement: The development of the search strategy was supported by Zoe Gibbs-Monaghan Liaison Librarian – Health and Life Sciences at the University of Liverpool. Ruaraidh Hill, Lecturer in evidence synthesis at the University of Liverpool provided beneficial insights into review's methodology.

Funding statement: Piotr Teodorowski is a PhD student supported by the National Institute for Health Research Applied Research Collaboration North West Coast (NIHR ARC NWC) Award number: 103. The views expressed in this publication are those of the author(s) and not necessarily those of the National Institute for Health Research or the Department of Health and Social Care.

Data statement: Data sharing not applicable as no datasets were created or analysed in this protocol

Competing interests: None declared

For peer review only

For peer review only - http://bmjopen.bmj.com/site/about/guidelines.xhtml

References

1. Mehta N, Pandit A. Concurrence of big data analytics and healthcare: A systematic review. *International Journal of Medical Informatics* 2018;114:57-65. doi: 10.1016/j.ijmedinf.2018.03.013
2. OpenSAFELY. OpenSAFELY: About Us 2021 [Available from: https://www.opensafely.org/about/ accessed 22 June 2021.
3. University of Liverpool. Children Growing Up in Liverpool (C-GULL), 2020.
4. Raghupathi W, Raghupathi V. Big data analytics in healthcare: promise and potential. *Health Information Science and Systems* 2014;2(1):3. doi: 10.1186/2047-2501-2-3
5. Zhang X, Pérez-Stable EJ, Bourne PE, et al. Big Data Science: Opportunities and Challenges to Address Minority Health and Health Disparities in the 21st Century. *Ethnicity & Disease* 2017;27(2):95. doi: 10.18865/ed.27.2.95
6. Lipworth W, Mason PH, Kerridge I, et al. Ethics and Epistemology in Big Data Research. *Journal of Bioethical Inquiry* 2017;14(4):489-500. doi: 10.1007/s11673-017-9771-3
7. Aitken M, de St. Jorre J, Pagliari C, et al. Public responses to the sharing and linkage of health data for research purposes: a systematic review and thematic synthesis of qualitative studies. *BMC Medical Ethics* 2016;17(1):73. doi: 10.1186/s12910-016-0153-x
8. Aitken M, Cunningham-Burley S, Pagliari C. Moving from trust to trustworthiness: Experiences of public engagement in the Scottish Health Informatics Programme. *Science and Public Policy* 2016;43(5):713-23. doi: 10.1093/scipol/scv075
9. Carter P, Laurie GT, Dixon-Woods M. The social licence for research: why care.data ran into trouble. *Journal of Medical Ethics* 2015;41(5):404. doi: 10.1136/medethics-2014-102374
10. Aitken M, Tully M, Porteous C, et al. Consensus Statement on Public Involvement and Engagement with Data-Intensive Health Research. *International Journal of Population Data Science* 2019;4 doi: 10.23889/ijpds.v4i1.586
11. Revell T. Google DeepMind's NHS data deal 'failed to comply' with law New Scientist2017 [Available from: https://institutions.newscientist.com/article/2139395-google-deepminds-nhs-data-deal-failed-to-comply-with-law/.
12. Taylor M. Information Governance as a Force for Good? Lessons to be Learnt from Care.data. *SCRIPTed* 2014;11 doi: 10.2966/scrip.110114.1
13. Hill EM, Turner EL, Martin RM, et al. "Let's get the best quality research we can": public awareness and acceptance of consent to use existing data in health research: a systematic review and qualitative study. *BMC Medical Research Methodology* 2013;13(1):72. doi: 10.1186/1471-2288-13-72
14. Data Saves Lives. What is Data Saves Lives? 2020 [Available from: https://datasaveslives.eu/aboutdsl accessed 11 January 2021.
15. Ford E, Boyd A, Bowles JKF, et al. Our data, our society, our health: A vision for inclusive and transparent health data science in the United Kingdom and beyond. *Learning Health Systems* 2019;3(3):e10191. doi: 10.1002/lrh2.10191
16. Beier K, Schweda M, Schicktanz S. Taking patient involvement seriously: a critical ethical analysis of participatory approaches in data-intensive medical research. *BMC Medical Informatics and Decision Making* 2019;19(1) doi: 10.1186/s12911-019-0799-7
17. Lalani M, Baines R, Bryce M, et al. Patient and public involvement in medical performance processes: A systematic review. *Health expectations : an international journal of public participation in health care and health policy* 2019;22(2):149-61. doi: 10.1111/hex.12852 [published Online First: 2018/12/11]
18. Ocloo J, Matthews R. From tokenism to empowerment: progressing patient and public involvement in healthcare improvement. *BMJ Quality & Safety* 2016;25(8):626-32. doi: 10.1136/bmjqs-2015-004839

For peer review only

For peer review only - http://bmjopen.bmj.com/site/about/guidelines.xhtml

19. Crawford MJ. Systematic review of involving patients in the planning and development of health care. *BMJ* 2002;325(7375):1263-63. doi: 10.1136/bmj.325.7375.1263
20. Mockford C, Staniszewska S, Griffiths F, et al. The impact of patient and public involvement on UK NHS health care: a systematic review. *International Journal for Quality in Health Care* 2012;24(1):28-38. doi: 10.1093/intqhc/mzr066
21. Sofolahan-Oladeinde Y, Newhouse RP, Lavallee DC, et al. Early assessment of the 10-step patient engagement framework for patient-centred outcomes research studies: the first three steps. *Family Practice* 2017;34(3):272-77. doi: 10.1093/fampra/cmx013
22. Stockdale J, Cassell J, Ford E. “Giving something back”: A systematic review and ethical enquiry into public views on the use of patient data for research in the United Kingdom and the Republic of Ireland. *Wellcome Open Research* 2019;3:6. doi: 10.12688/wellcomeopenres.13531.2
23. Kalkman S, Van Delden J, Banerjee A, et al. Patients’ and public views and attitudes towards the sharing of health data for research: a narrative review of the empirical evidence. *Journal of Medical Ethics* 2019:medethics-2019-. doi: 10.1136/medethics-2019-105651
24. Howe N, Giles E, Newbury-Birch D, et al. Systematic review of participants’ attitudes towards data sharing: a thematic synthesis. *Journal of Health Services Research & Policy* 2018;23(2):123-33. doi: 10.1177/1355819617751555
25. Rohwer A, Pfadenhauer L, Burns J, et al. Series: Clinical Epidemiology in South Africa. Paper 3: Logic models help make sense of complexity in systematic reviews and health technology assessments. *Journal of Clinical Epidemiology* 2017;83:37-47. doi: 10.1016/j.jclinepi.2016.06.012
26. Gough D, Thomas J, Oliver S. Clarifying differences between review designs and methods. *Systematic Reviews* 2012;1(1):28. doi: 10.1186/2046-4053-1-28
27. Arksey H, O'Malley L. Scoping studies: towards a methodological framework. *International Journal of Social Research Methodology* 2005;8(1):19-32. doi: 10.1080/1364557032000119616
28. Colquhoun HL, Levac D, O'Brien KK, et al. Scoping reviews: time for clarity in definition, methods, and reporting. *Journal of Clinical Epidemiology* 2014;67(12):1291-94. doi: 10.1016/j.jclinepi.2014.03.013
29. Levac D, Colquhoun H, O'Brien KK. Scoping studies: advancing the methodology. *Implementation Science* 2010;5(1):69. doi: 10.1186/1748-5908-5-69
30. Munn Z, Peters MDJ, Stern C, et al. Systematic review or scoping review? Guidance for authors when choosing between a systematic or scoping review approach. *BMC Medical Research Methodology* 2018;18(1) doi: 10.1186/s12874-018-0611-x
31. Moher D, Shamseer L, Clarke M, et al. Preferred reporting items for systematic review and meta-analysis protocols (PRISMA-P) 2015 statement. *Systematic Reviews* 2015;4(1):1. doi: 10.1186/2046-4053-4-1
32. Dawson S, Campbell SM, Giles SJ, et al. Black and minority ethnic group involvement in health and social care research: A systematic review. *Health Expectations* 2018;21(1):3-22. doi: 10.1111/hex.12597
33. Harrison JD, Auerbach AD, Anderson W, et al. Patient stakeholder engagement in research: A narrative review to describe foundational principles and best practice activities. *Health Expectations* 2019;22(3):307-16. doi: 10.1111/hex.12873
34. Islam S, Small N. An annotated and critical glossary of the terminology of inclusion in healthcare and health research. *Research Involvement and Engagement* 2020;6(1):14. doi: 10.1186/s40900-020-00186-6
35. INVOLVE. What is public involvement in research? 2020 [Available from: https://www.invo.org.uk/find-out-more/what-is-public-involvement-in-research-2/ accessed 21 September 2020.

For peer review only - http://bmjopen.bmj.com/site/about/guidelines.xhtml

36. Thomas J, Harden A. Methods for the thematic synthesis of qualitative research in systematic reviews. *BMC Medical Research Methodology* 2008;8(1):45. doi: 10.1186/1471-2288-8-45
37. Hong Q, Pluye P, Fàbregues S, et al. Mixed Methods Appraisal Tool (MMAT) 2018 [Available from: http://mixedmethodsappraisaltoolpublic.pbworks.com/w/file/fetch/127916259/MMAT_2018_criteria-manual_2018-08-01_ENG.pdf accessed 12/11 2020.
38. Lewin S, Glenton C, Munthe-Kaas H, et al. Using Qualitative Evidence in Decision Making for Health and Social Interventions: An Approach to Assess Confidence in Findings from Qualitative Evidence Syntheses (GRADE-CERQual). *PLOS Medicine* 2015;12(10):e1001895. doi: 10.1371/journal.pmed.1001895
39. Cottrell EK, Whitlock EP, Kato E, et al. Defining the benefits and challenges of stakeholder engagement in systematic reviews. *Comparative Effectiveness Research* 2015;5:13-19. doi: http://dx.doi.org/10.2147/CER.S69605
40. INVOLVE. Public involvement in systematic reviews: Supplement to the briefing notes for researchers. Eastleigh: INVOLVE, 2012.
41. Staniszewska S, Brett J, Simera I, et al. GRIPP2 reporting checklists: tools to improve reporting of patient and public involvement in research. *BMJ* 2017:j3453. doi: 10.1136/bmj.j3453
42. Mouton Dorey C, Baumann H, Biller-Andorno N. Patient data and patient rights: Swiss healthcare stakeholders' ethical awareness regarding large patient data sets - a qualitative study. *BMC medical ethics* 2018;19(1):20-20. doi: 10.1186/s12910-018-0261-x

For peer review only

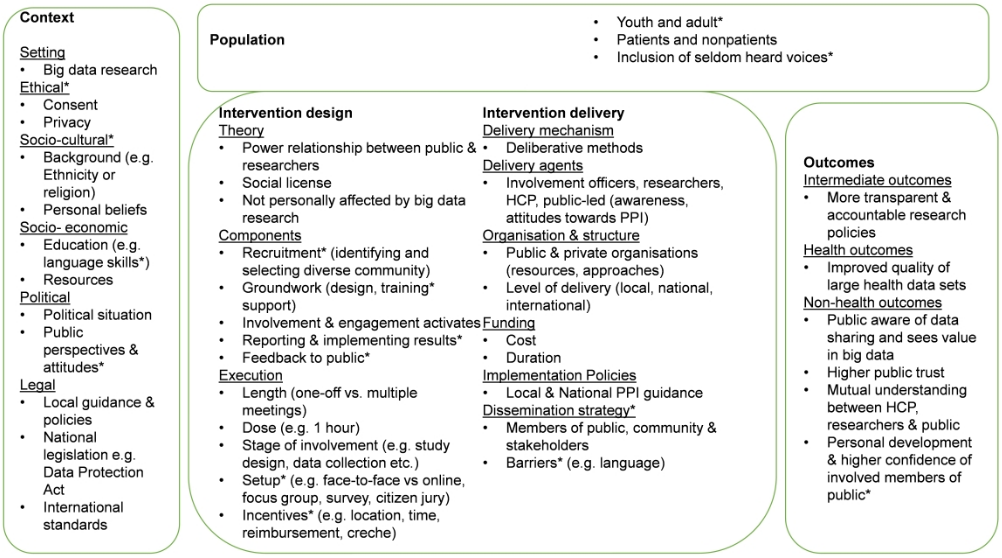

Figure 1 System logic model of public involvement and engagement in big data research.

677x381mm (300 x 300 DPI)

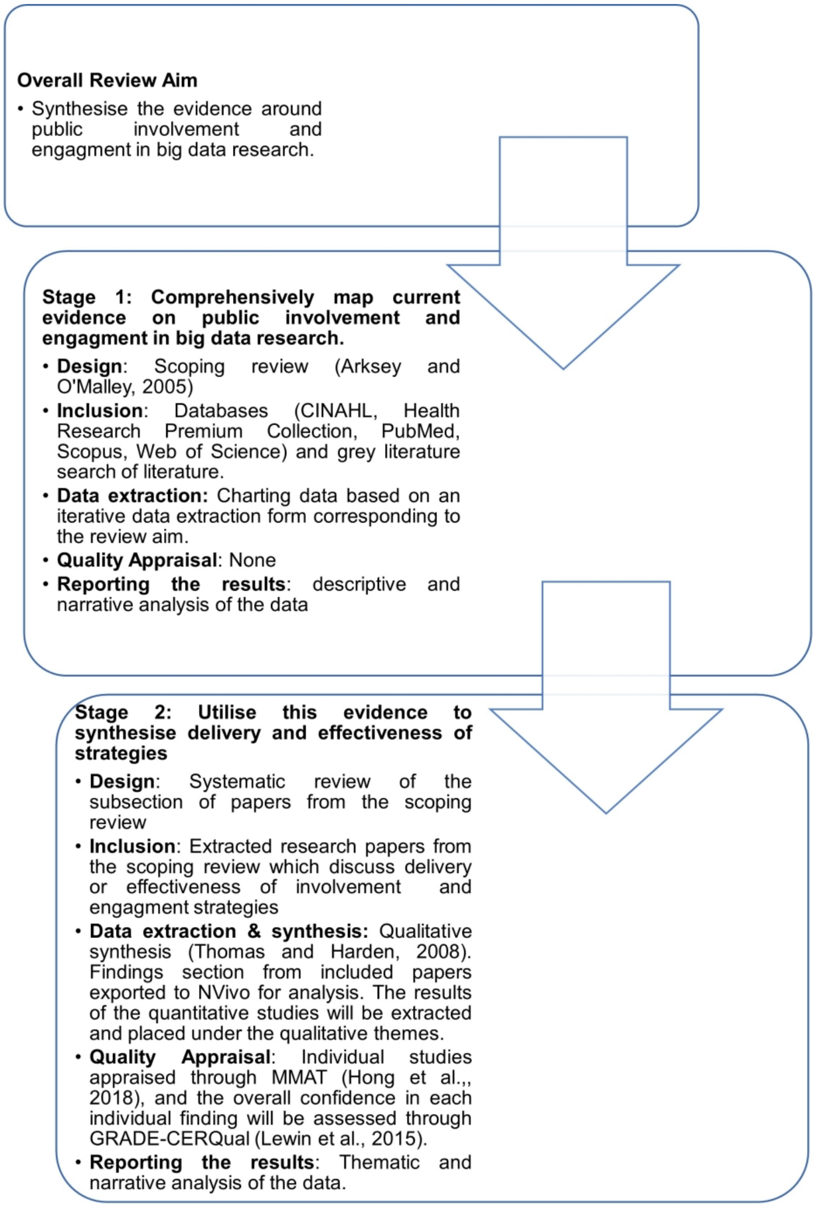

Figure 2 Systematic map of the review process

175x258mm (330 x 330 DPI)

Preferred Reporting Items for Systematic reviews and Meta-Analyses extension for Scoping Reviews (PRISMA-ScR) Checklist

SECTION	ITEM	PRISMA-ScR CHECKLIST ITEM	REPORTED ON PAGE #
**TITLE**			
Title	1	Identify the report as a scoping review.	Page 2, lines 1-3
**ABSTRACT**			
Structured summary	2	Provide a structured summary that includes (as applicable): background, objectives, eligibility criteria, sources of evidence, charting methods, results, and conclusions that relate to the review questions and objectives.	Page 2, lines 5-24
**INTRODUCTION**			
Rationale	3	Describe the rationale for the review in the context of what is already known. Explain why the review questions/objectives lend themselves to a scoping review approach.	Page 2, line 43 to Page 3, line-54
Objectives	4	Provide an explicit statement of the questions and objectives being addressed with reference to their key elements (e.g., population or participants, concepts, and context) or other relevant key elements used to conceptualize the review questions and/or objectives.	Page 4, lines 15-21 Page 5, line 12- Page 6, line 33
**METHODS**			
Protocol and registration	5	Indicate whether a review protocol exists; state if and where it can be accessed (e.g., a Web address); and if available, provide registration information, including the registration number.	n/a
Eligibility criteria	6	Specify characteristics of the sources of evidence used as eligibility criteria (e.g., years considered, language, and publication status), and provide a rationale.	Page 5, line 12- Page 6, line 33
Information sources*	7	Describe all information sources in the search (e.g., databases with dates of coverage and contact with authors to identify additional sources), as well as the date the most recent search was executed.	Page 4, line 41 to Page 5, line 8
Search	8	Present the full electronic search strategy for at least 1 database, including any limits used, such that it could be repeated.	Page 5, line 10
Selection of sources of evidence†	9	State the process for selecting sources of evidence (i.e., screening and eligibility) included in the scoping review.	Page 6, lines 37- 45
Data charting process‡	10	Describe the methods of charting data from the included sources of evidence (e.g., calibrated forms or forms that have been tested by the team before their use, and whether data charting was done independently or in duplicate) and any processes for obtaining and confirming data from investigators.	Page 6, line 47 to Page 7, line 11
Data items	11	List and define all variables for which data were sought and any assumptions and simplifications made.	n/a
Critical appraisal of individual sources of evidence§	12	If done, provide a rationale for conducting a critical appraisal of included sources of evidence; describe	Page 7, lines, 34- 40.

For peer review only - http://bmjopen.bmj.com/site/about/guidelines.xhtml

SECTION	ITEM	PRISMA-ScR CHECKLIST ITEM	REPORTED ON PAGE #
		the methods used and how this information was used in any data synthesis (if appropriate).	
Synthesis of results	13	Describe the methods of handling and summarizing the data that were charted.	Page 7, line 14-16
**RESULTS**			
Selection of sources of evidence	14	Give numbers of sources of evidence screened, assessed for eligibility, and included in the review, with reasons for exclusions at each stage, ideally using a flow diagram.	n/a
Characteristics of sources of evidence	15	For each source of evidence, present characteristics for which data were charted and provide the citations.	n/a
Critical appraisal within sources of evidence	16	If done, present data on critical appraisal of included sources of evidence (see item 12).	n/a
Results of individual sources of evidence	17	For each included source of evidence, present the relevant data that were charted that relate to the review questions and objectives.	n/a
Synthesis of results	18	Summarize and/or present the charting results as they relate to the review questions and objectives.	n/a
**DISCUSSION**			
Summary of evidence	19	Summarize the main results (including an overview of concepts, themes, and types of evidence available), link to the review questions and objectives, and consider the relevance to key groups.	n/a
Limitations	20	Discuss the limitations of the scoping review process.	Page 8 line 3-9
Conclusions	21	Provide a general interpretation of the results with respect to the review questions and objectives, as well as potential implications and/or next steps.	n/a
**FUNDING**			
Funding	22	Describe sources of funding for the included sources of evidence, as well as sources of funding for the scoping review. Describe the role of the funders of the scoping review.	Page 9, line 10-14

JBI = Joanna Briggs Institute; PRISMA-ScR = Preferred Reporting Items for Systematic reviews and Meta-Analyses extension for Scoping Reviews.
* Where *sources of evidence* (see second footnote) are compiled from, such as bibliographic databases, social media platforms, and Web sites.
† A more inclusive/heterogeneous term used to account for the different types of evidence or data sources (e.g., quantitative and/or qualitative research, expert opinion, and policy documents) that may be eligible in a scoping review as opposed to only studies. This is not to be confused with *information sources* (see first footnote).
‡ The frameworks by Arksey and O'Malley (6) and Levac and colleagues (7) and the JBI guidance (4, 5) refer to the process of data extraction in a scoping review as data charting.
§ The process of systematically examining research evidence to assess its validity, results, and relevance before using it to inform a decision. This term is used for items 12 and 19 instead of "risk of bias" (which is more applicable to systematic reviews of interventions) to include and acknowledge the various sources of evidence that may be used in a scoping review (e.g., quantitative and/or qualitative research, expert opinion, and policy document).

From: Tricco AC, Lillie E, Zarin W, O'Brien KK, Colquhoun H, Levac D, et al. PRISMA Extension for Scoping Reviews (PRISMAScR): Checklist and Explanation. Ann Intern Med. 2018;169:467–473. doi: 10.7326/M18-0850.

For peer review only
